# ART coverage and viral suppression among female sex workers living with HIV in eThekwini, South Africa: Baseline findings from the Siyaphambili study

**DOI:** 10.1371/journal.pgph.0002783

**Published:** 2024-05-22

**Authors:** Carly A. Comins, Stefan Baral, Mfezi Mcingana, Lily Shipp, Deliwe Rene Phetlhu, Katherine Young, Vijayanand Guddera, Harry Hausler, Sheree Schwartz

**Affiliations:** 1 Department of Epidemiology, Johns Hopkins Bloomberg School of Public Health, Baltimore, Maryland, United States of America; 2 TB HIV Care, Cape Town, South Africa; 3 Department of Nursing, Sefako Makgatho Health Sciences University, Pretoria, South Africa; 4 Research and Innovation, heaps.ai, Bengaluru, India; 5 University of Pretoria, Pretoria, South Africa; University of the Witwatersrand, SOUTH AFRICA

## Abstract

In South Africa >60% of female sex workers (FSW) are living with HIV, the majority of whom are not virally suppressed. Identifying multi-level determinants of viral suppression is central to developing implementation strategies to promote retention in HIV care and viral suppression among FSW with unmet treatment needs. Adult cisgender FSW living with HIV for ≥6 months, conducting sex work as their primary source of income, and residing in Durban (South Africa) were enrolled into the Siyaphambili Study, a sequential multiple assignment randomized trial. Baseline viral load and CD4 were assessed, and an interviewer-administered survey was conducted, capturing socio-demographic, reproductive and sexual history and behaviors, vulnerabilities, substance use, mental health, and stigma. We assessed baseline determinants of viral suppression (<50 copies/mL) using bivariate and multivariable robust poisson regression, considering associations across the individual, network, environmental and macrostructural levels. From June 2018 –March 2020, 1,644 women were screened, with 1,391 eligible FSW living with HIV enrolled. The analyses were conducted among the 1,373 participants with baseline data. Overall, 65% (889/1,373) of participants were reported to be on antiretroviral therapy and 38% (520/1,373) were virally suppressed. In the multivariable model, FSW who experienced a lack of housing in the prior six months were less likely to be virally suppressed (aPR: 0.72, 95%CI 0.56–0.91), while older FSW (aPR: 1.46 95%CI: 1.16–1.83 for 30–39 years old vs. 18–29 years old; aPR: 2.15 95%CI: 1.64–2.80 for 40+ years vs. 18–29 years old) and FSW reporting hormonal or long-acting contraception use were more likely to be virally suppressed (aPR: 1.19 95% CI: 1.00–1.43). We found vulnerability to be high among FSW living with HIV in South Africa and identified individual and structural determinants associated with viral suppression. Taken together these results suggest optimizing HIV treatment outcomes necessitates supporting younger sex workers and addressing housing instability.

**Trial registration**: NCT03500172.

## Introduction

About one fifth of the world’s population living with HIV is estimated to live in South Africa [1]. While South Africa is considered to have a generalized HIV epidemic, there have been sustained disparities in HIV incidence and HIV treatment outcomes [1]. Unmet HIV prevention and treatment needs continue to place female sex workers (FSW) at increased risk for HIV acquisition, HIV-related morbidity and mortality, and onward HIV transmission. There are an estimated 146,000 FSW working in South Africa and 62% are estimated to be living with HIV [2, 3]. Data on retention in HIV care and viral suppression among FSW is limited; but when available, treatment outcomes are suboptimal [4–8]. Moreover, modeling estimates that 40% of new HIV infections in South Africa are among key populations, including FSW, and their sexual partners [9]. For FSW specifically, incident infections are predominantly among younger FSW, and younger FSW living with HIV have consistently lower levels of engagement in HIV care and treatment [10].

FSW living with HIV experience diverse HIV treatment outcomes and needs. Formative qualitative research among FSW in eThekwini (Durban), South Africa identified individual-, network-, community-, and policy-level barriers to antiretroviral therapy (ART) initiation, retention, and adherence per Baral et al.’s socioecological model [11]. Emergent individual-level barriers included age, acceptance of HIV status, heavy substance use, transportation challenges, food insecurity, pill burden, motivation, and psychosocial barriers [11–13]. These individual-level barriers influencing treatment access and adherence have been consistently observed among FSW in South Africa and in other settings [13–15]. The relevance of network- and community-level barriers center around sex work venue dynamics. At indoor sex work venues (i.e., brothels, private homes, guest houses), themes identified to impact ART initiation, retention, and adherence included non-disclosure of HIV status to brothel managers, costs to reserve rooms while attending the clinic, and sabotage from co-workers based on HIV status. At outdoor venues (i.e., streets, parks, beaches, truck stops), FSW expressed challenges to ART storage. Other relevant network-related factors influencing access and sustained use that have been identified include ART theft, physical and sexual violence, and spontaneous client demands/opportunities [11, 16, 17]. Additionally, while FSW working during the day face the choice of visiting the clinic for treatment or selling sex, qualitative research also highlighted that FSW working at night face challenges accessing the clinic during regular daytime hours due to fatigue or working the following night due to fatigue [11, 17].

Further, in addition to known individual and inter-personal factors that have been quantitatively assessed [14, 18–24], the central role of structural determinants has been increasingly recognized, although less studied [25, 26]. Structural determinants are external contextual factors that shape HIV acquisition and transmission among FSW [25], heterogeneously impacting FSW by mitigating or potentiating HIV risks and vulnerabilities [25, 26]. For FSW living with HIV, structural drivers can occur across levels and can include but are not limited to the criminalization of sex work, policing practices, sex work- and HIV-related stigmas, clinic procedures and ART stock, mobility, and housing—all of which occur and interrelate across various levels and influence individual and interpersonal factors [8, 12, 25–32].

Determinants of HIV treatment use and viral suppression have been identified among South African women living with HIV and FSW engaged in sex-work programs [14, 33–35], but limited data characterizing the multi-level determinants of viral suppression among South African FSW exist. The objective of this to describe HIV treatment outcomes and to assess individual-, sexual network-, work and social environment-, and macrostructural-level factors related to viral suppression among a large cohort of FSW living with HIV in eThekwini, South Africa. The results of this work are intended to inform HIV treatment programming and delivery efforts for FSW living with HIV.

## Methods

### Study overview

This analysis utilized baseline data from FSW living with HIV enrolled in the Siyaphambili Study, a pragmatic trial to assess the effectiveness and efficiency of HIV treatment support strategies implemented over an 18-month period [36]. The study was conducted within TB HIV Care, an existing HIV program in the eThekwini Metropolitan Municipality of KwaZulu-Natal. The South African non-profit TB HIV Care program branch in eThekwini (Durban) has been serving FSW since 2012, offering HIV prevention methods (i.e., condoms, pre-exposure prophylaxis), voluntary counseling and testing, and ART treatment initiation and management at their drop-in-center. All service provision is in accordance with the South Africa’s standard of HIV care and treatment put forth in the Department of Health National Guidelines [37, 38]. Further, the program employs FSW peers to support HIV prevention activities, linkage to care, and ART retention, and maintains strong relationships with the local FSW community through community engagement, participation, and partnership.

### Study population

The Siyaphambili trial focused on assessing the impact of HIV treatment strategies aimed to support engagement in HIV care and viral suppression among FSW whose HIV treatment needs were not met by the national HIV programs [i.e., defined as not being virally suppressed six months after HIV diagnosis]. Siyaphambili protocol procedures, including power and sample size calculations, recruitment, and data collection, have been described in detail [36]. Briefly, all interested FSW were assessed for eligibility via a phased screening process based on inclusion and exclusion criteria including rapid HIV and pregnancy testing. Adult cisgender women living with HIV for at least six months, engaging in sex work as their primary source of income and living and residing in Durban were eligible for recruitment. ART-naïve individuals, as well as those on first line ART regimens for two or more months, were eligible (FSW newly initiating ART, within the prior 2 months, were ineligible as there was not yet sufficient time to virally suppress). FSW pregnant or participating in an adherence group at the time of recruitment were not eligible. FSW peers, in addition to the TB HIV Care program peers, led the research team in recruitment efforts utilizing standardized recruitment scripts, branded recruitment flyers, and business cards from the mobile van, sex work venues, or the TB HIV Care drop-in center. The trial aimed to randomize non-virally suppressed women into an intervention, and thus recruitment prioritized high volume sites served by the TB HIV Care treatment program. Despite ranking by order of potential eligibility, over time, all sites where the program was implementing services that could safely be accessed by the research team were visited. Recruitment at sex work venues utilized convenience sampling, consecutively recruiting, and offering eligibility screening to all potentially FSW present (i.e., non-probabilistic sampling). Recruitment continued through the ranked venues until enrollment targets were reached.

### Sample size

The Siyaphambili trial was tailored to FSW living with HIV who were non-virally suppressed at baseline, and therefore, the targeted sample size for trial randomization was 782 non-virally suppressed participants, rounded to 800 to safeguard against loss-to-follow-up (400 per arm) [36]. Based on local estimates of viral suppression among FSW to be 30%, 1,905 FSW living with HIV were estimated to be screened at enrollment in order to obtain the targeted sample size for randomization into the trial [36]. With 1,644 screened, 1,391 enrolled, and 520 virally suppressed, we are well powered to explore associations with viral suppression.

### Study enrollment and data collection

Biometric iris scanning provided a unique 12-digit study identification number to all participants enrolled [39]. The unique identification number, generated through a scan of the participant’s irises, was used to identify the participant and to link biological sample results and survey questions. Following enrollment and identification via the iris scanner, 5 mL of whole blood per participant was obtained by a nurse for baseline CD4 count and viral load testing. Following the blood draw, a baseline survey was administered in-person in Zulu or English by a trained quantitative data collector. The baseline survey contained the following modules: demographic and socioeconomic characteristics; personal and sexual history, reproductive health and health seeking behaviors, physical and sexual vulnerability, quality of life, alcohol and drug use, social support and mental health, stigma, and resilience. Participants received 100 ZAR (~ 7 USD) reimbursement for completion of the baseline study visit.

### Variables and outcomes

All examined covariates were self-reported, and variables for inclusion in the bivariate and multivariable analyses were identified per Shannon et al.’s Structural Determinants Framework for HIV and Sex Work [26]. We utilized this framework to organize our results as depicted in **S1 Fig**. Viral suppression, the primary outcome, was defined as <50 copies/mL per the South African National Treatment Guidelines [40, 41].

#### Individual-level factors

Behavioral factors considered included age at first exchange of sex for money (in years), contraception use, current relationship status (steady partner cohabitating/steady partner living apart/single), heavy alcohol use, drug use in the prior month, history of injection drug use. Contraception use included current use of a hormonal/long-acting contraception method (i.e., oral birth control, intrauterine device, injectable birth control, and the implant). Heavy alcohol was enumerated from drinking ≥5 alcoholic beverages at least twice weekly [42]. Drug use included marijuana, cocaine (or “rice drug”), crack (or “rock” or “tear drop”), methamphetamine (or “Crystal”), ecstasy (“Mercedes drug”), heroin, and heroin derivatives “whoonga” and “sugar” [43, 44]. Biological factors of FSW living with HIV included age, depressive symptoms, pregnancy history (≥ 1 prior pregnancy), STI diagnosis or symptoms in the prior six months, and duration of HIV infection (in years). Mental health was assessed by screening for depressive symptoms using the 9-item Patient Health Questionnaire depression module [PHQ-9] [45]. Depressive symptoms were dichotomized per established PHQ-9 cut-points as moderate to severe depression and no to mild depression. Other clinical factors assessed included the CD4 count at study enrollment.

#### Sexual network-level factors

Sexual network factors assessed were the number of sexual clients and/or partners in the prior month, consistent condom use, and the frequency of sex work in the prior month. Consistent condom use was defined as always using a condom in the prior month and was reported among new clients, regular clients, and non-paying partners. To assess the frequency of sex work, participants reported the average number of days per month in which they sell sex. The response was dichotomized at 20 days or two-thirds of the prior month, to reflect full time employment.

#### Work and social environment-level factors

Factors related to the work and social environments included the primary location of sex work (indoor/outdoor sex work venues), forced shared earnings from sex work (yes/no), carrying condoms during sex work (yes/no), historical experiences of physical and sexual violence (yes/no), and perceived safety in daily life. Participants were categorized based on where they primarily had sex. Outdoor sex work venues included the street, park, public garden, beach, cemetery, or private vehicle, while indoor sex work venues included the private home, brothel, bar, private party, hotel or guest house, or shelter. A binary variable was created to capture whether participants reported being forced to share their earnings from sex work with another individual (i.e., with a pimp, brothel owner, police, or drug lord). Any history of physical violence included ever being pushed, shoved, slapped, hit, kicked, choked, or otherwise physically hurt. Any history of sexual violence included ever being forced to have sex. Perceived safety in daily life was dichotomized as no safety/some degree of safety.

#### Macrostructural-level factors

Macrostructural factors measured included HIV- and sex work-related stigmas, housing, number of household residents, enough money to meet needs, number of dependents financially supported by sex work, and mobility. Enacted and anticipated sex work-related and HIV stigmas were initially developed through literature review and stakeholder engagement, with adaptation in several settings [46–48] and reliability of sex work-related items demonstrated [49]. Enacted stigma included the experience of discrimination and/or stereotyping from others due to sex work or HIV status and comprised of 10 closed (yes/no) items for sex work-related stigma and seven closed items for HIV-related stigma. Anticipated stigma included the expectation of discrimination and/or stereotyping from others due to sex work or HIV status and comprised of five closed items for sex work-related stigma and four closed items for HIV-related stigma. Enacted and anticipated stigmas were categorized as no stigma or any stigma (“yes” to at least one item) [50]. Participants’ experience with housing in the prior six months were categorized as unhoused (living in a shelter or with no place to live) or housed. The number of other people the participant reported living with was dichotomized at the median of 3 people. Enough money to meet needs was dichotomized as not having enough money to meet daily needs all of the time vs. typically having enough money to meet daily needs. Dependents included any individual (i.e., adult or child) financially supported by the participant’s sex work. We dichotomized the number of dependents at the median of 2. Finally, mobility in the prior six months included any travel (>1 day) outside of Durban for sex work or another reason.

### Data analysis

Data were collected, managed, and securely stored using REDCap^TM^ and analyzed in STATA version 15.0 [51–53]. Descriptive statistics were conducted across variables and the unadjusted bivariate associations between risk factors and viral suppression were assessed using Pearson’s chi-squares, Fisher’s exact tests, and t-tests. Risk factors examined were identified per a modification of Shannon et al.’s Structural Determinant’s Framework for HIV and Sex Work [26], and factors for inclusion in the multivariable models were determined based on a bivariate analysis p-value of <0.1. Modified poisson regression models were used to estimate the prevalence ratios (PR) and adjusted prevalence ratios (aPRs) for the associations between risk factors and viral suppression. Missingness was addressed via Multiple Imputation using Chained Equations [54].

### Ethics

The Siyaphambili study and research protocol was approved by the Johns Hopkins School of Public Health Institutional Review Board in the United States, the University of the Western Cape Biomedical Research Ethics Committee in South Africa, and the eThekwini Municipality and KwaZulu-Natal Provincial Departments of Health. All participants provided written informed consent.

## Results

**Fig 1:**
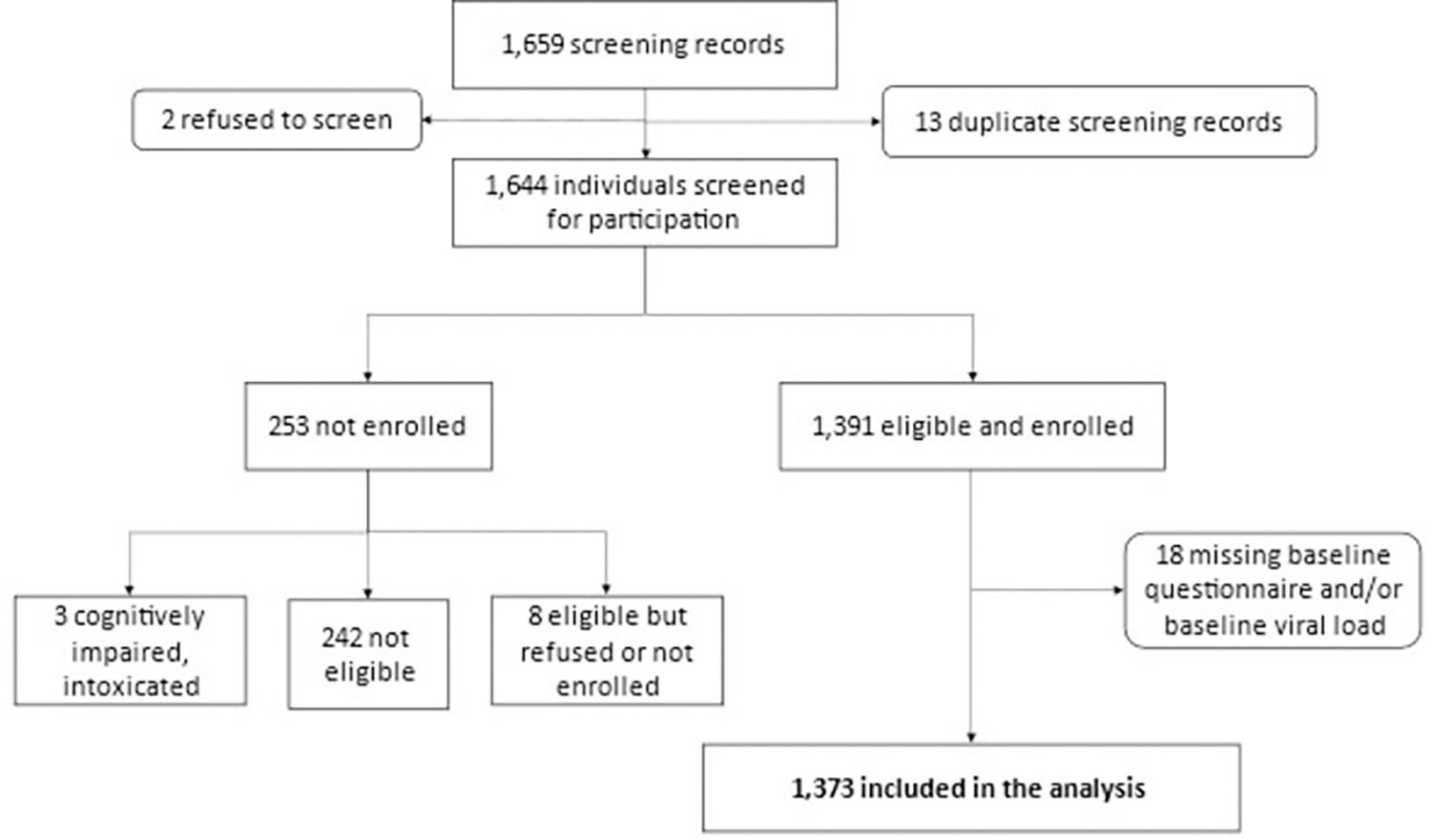
Flow diagram of enrollment into the Siyaphambili study between June 2018 and March 2020 in eThekwini, South Africa.

A total of 1,644 women were screened for participation, of which 1,391 (84.6%) were enrolled into the Siyaphambili study from June 22, 2018 –March 23, 2020 (**Fig 1**). Of the 15.4% (253/1,644) women not enrolled, 95.6% (242/253) were not eligible, 3.2% (8/253) were eligible but refused participation, and 1.2% (3/253) were intoxicated and unable to screen for eligibility or provide informed consent. The main reason for ineligibility was HIV status (61.6%, 149/242). Reasons for ineligibility are depicted in **S2 Fig**.

### Demographic characteristics

Overall, baseline data were available for 1,379/1,391 (99.1%); missing questionnaires were not completed due to participant time constraints or technologic failures of the tablet used for data collection. Baseline viral load data were available for 1,380/1,391 (99.2%); missing baseline viral loads were either not completed or the sample was unable to be processed by the laboratory (i.e., hemolyzed or insufficient). The analysis was conducted among participants with baseline data (i.e., questionnaire and viral load data; n = 1,373).

The median age of Siyaphambili participants was 31 years (interquartile range [IQR]: 27–37), ranging from 18–65 years old (**Table 1**). The majority of participants were Black (97.2%) and South African (98.2%). Almost all (97.6%, 1,340/1,373) women could read and/or write. The majority of participants received some formal schooling (98.2%, 1,348/1,373) and a few FSW (n = 13) reported current enrollment in school. A total of 43.3% (593/1,373) reported being an orphan (i.e., no living parents). Half of all participants were single, while 15.2% (209/1,373) cohabitated with a steady partner and 34.1% (468/1,373) lived separately from a steady partner.

**Table 1:** Characteristics of female sex workers living with HIV in the Siyaphambili study in eThekwini, South Africa, stratified by viral suppression (N = 1,373).

Variable	Total, n (%) (n = 1,373)	Not virally Suppressed, n (%) (n = 853)	Virally suppressed, n (%) (n = 520)	p-value
**Demographic**				
Age				
18–29 years	570 (41.5%)	431 (50.5%)	139 (26.7%)	<0.001
30–39 years	558 (40.6%)	330 (38.7%)	228 (43.9%)	
40+ years	245 (17.9%)	92 (10.8%)	153 (29.4%)	
Education				
None	25 (1.8%)	11 (1.3%)	14 (2.7%)	0.171
Incomplete secondary school or lower	1,086 (79.1%)	680 (79.7%)	406 (78.1%)	
Complete secondary education or higher	262 (19.1%)	162 (19.0%)	100 (19.2%)	
Experienced lack of housing in prior 6 months ^a^	404 (29.5%)	299 (35.2%)	105 (20.2%)	<0.001
**Clinical**				
Duration of HIV infection (years), median (IQR) ^b^	4.5 (1.7,9.1)	3.8 (1.4,8.6)	5.8 (2.4,10.2)	<0.001
CD4, median (IQR) ^c^	513 (319,749)	408 (246,608)	679 (513,901)	<0.001
STI diagnosis or symptoms in prior 6 months	435 (31.7%)	296 (34.7%)	139 (26.7%)	0.002
≥1 Pregnancy ^d^	1,173 (85.6%)	712 (83.6%)	461 (88.7%)	0.007
Use of hormonal/long-acting contraception ^d^^,^^e^	483 (35.2%)	277 (32.5%)	206 (39.6%)	0.007
Moderate to severe depressive symptoms ^f^	458 (33.8%)	296 (35.7%)	162 (31.1%)	0.195
**Behavioral**				
Age at first exchange of sex for money (years), median (IQR) ^g^	23 (20,27)	22 (19,26)	24 (20,29)	<0.001
Primarily have sex with clients in outdoor venues ^h^	364 (26.5%)	223 (26.1%)	141 (27.1%)	0.659
Average number of days selling sex per month, median (IQR)	20 (13, 30)	20 (14, 30)	20 (12, 26)	<0.001
Alcohol use (5+ daily drinks ≥2x weekly)	481 (35.0%)	307 (36.0%)	174 (33.5%)	0.341
Drug use in prior 30 days ^i^^,^^j^	795 (57.9%)	549 (64.4%)	246 (47.3%)	<0.001
Prior injection drug use ^k^	55 (4.0%)	46 (5.2%)	9 (1.7%)	0.001
History of physical violence ^d^	740 (53.9%)	491 (57.6%)	249 (47.9%)	0.001
History of sexual violence ^h^	515 (37.5%)	360 (42.3%)	155 (29.8%)	<0.001
Consistent condom use with new clients in prior month ^l^	818 (60.4%)	493 (58.9%)	325 (62.9%)	0.290
Consistent condom use with regular clients in prior month ^m^	678 (50.2%)	405 (48.4%)	273 (53.0%)	0.161
Consistent condom use with nonpaying partners in prior month ^n^	168 (12.4%)	100 (11.8%)	68 (13.2%)	0.559
Mobility outside Durban (>1 day in prior six months) ^o^	158 (11.7%)	99 (11.8%)	59 (11.5%)	0.868

^a^ missing n = 4

^b^ missing n = 34

^c^ missing n = 78

^d^ missing n = 2

^e^ Hormonal / long-acting contraception includes birth control pill, intrauterine device, injectable birth control, implant.

^f^ missing n = 17

^g^ missing n = 6

^h^ missing n = 3

^I^ missing n = 27

^j^ Drug use includes marijuana, cocaine, crack, methamphetamine, ecstasy, heroin, and heroin derivatives.

^k^ missing n = 6

^l^ missing n = 19

^m^ missing n = 22

^n^ missing n = 14

^o^ missing n = 21

History of sex work varied across women. On average, women had been engaged in sex work (i.e., sex in exchange for money or good) for a median of 6.5 years [range 0–36 years]. When asked the main reason for starting to sell sex, the majority indicated the need to feed themselves or family or reported that sex work was the best opportunity available to them (54.8%, 753/1,373; 30.3%, 416/1,373, respectively); 94.4% (1,296/1,373) reported no other forms of employment. Almost all (93.7%, 1,297/1,373) sold sex exclusively in Durban during the preceding six months and few used the internet to sell sex or meet clients (3.9%, 53/1,373). Disclosure of sex work to a family or partner was limited (28.0%, 384/1,373 and 38.6% 274/710, respectively). The median number of clients/partners in the past month was 18 (IQR 10, 31).

### HIV treatment

**Fig 2:**
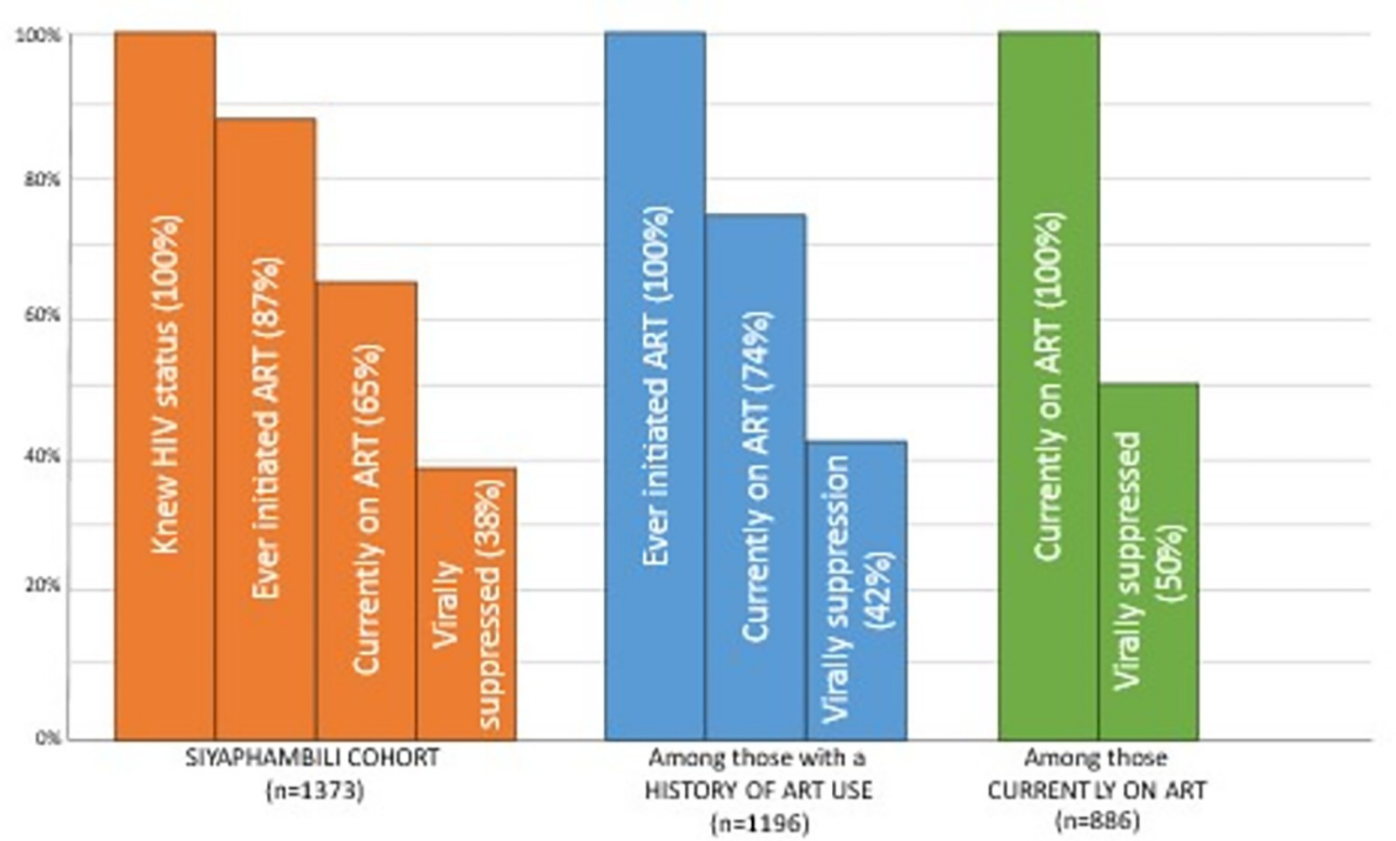
Modified HIV treatment cascade among female sex workers living with HIV and enrolled in the Siyaphambili study between June 2018 and March 2020 in eThekwini, South Africa (n = 1,373).

All Siyaphambili participants reported knowing their HIV status at enrollment; 14% were recently diagnosed (<1 year). The majority of women had previously initiated ART (87.1%, 1,196/1,373), 64.5% (886/1,373) reported currently taking treatment, and 37.9% (520/1,373) were confirmed by laboratory results to be virally suppressed (**Fig 2**). Of those currently on ART, 26.2% (231/883; 3 did not respond) obtained their treatment from TB HIV Care and 48.8% (422/864; 22 did not respond) self-reported 100% adherence in the prior month.

### Factors associated with viral suppression

Numerous factors across the individual-, sexual network-, work and social environments-, and macrostructural-level were found to be associated with viral suppression in bivariate analyses (**Table 2**). In the bivariate models, variables positively associated with viral suppression included age, prior pregnancy, duration of HIV infection, hormonal/long-acting contraception use, some degree of perceived safety, and number of dependents reliant on sex work. Variables associated with not being virally suppressed in the bivariate analyses included STI diagnosis or symptoms in the prior six months, drug use in the prior 30 days, sex work frequency per month, history of physical violence, history of sexual violence, experienced lack of housing, number of household residents, and anticipated sex work-related stigma. In the multivariable model, FSW who were unhoused in the prior six months were less likely to be virally suppressed (aPR: 0.72, 95%CI 0.56–0.91), while older FSW (aPR: 1.46 95%CI: 1.16–1.83 for 30–39 years old vs. 18–29 years old; aPR: 2.15 95%CI: 1.64–2.80 for 40+ years vs. 18–29 years old) and FSW reporting hormonal or long-acting contraception use were more likely to be virally suppressed (aPR: 1.19 95% CI: 1.00–1.43).

**Table 2:** Correlates of viral suppression among female sex workers enrolled in the Siyaphambili study between June 2018 and March 2020 in eThekwini, South Africa (n = 1,373).

Variable	Bivariable		Multivariable	
	PR	95% CI	aPR	95% CI
**INDIVIDUAL**				
**Biological**				
Age				
18–29 years	REF	REF	REF	REF
30–39 years	**1.68**	**1.40–2.00**	**1.46**	**1.16–1.83**
40+ years	**2.56**	**2.15–3.04**	**2.15**	**1.64–2.80**
Depressive symptoms				
None/mild depression	REF	REF		
Moderately to severe depression	0.91	0.76–1.10		
History of pregnancy				
Never pregnant	REF	REF	REF	REF
≥1 Prior pregnancy	**1.33**	**1.02–1.75**	0.93	0.70–1.24
STI diagnosis or symptoms in prior 6 months				
No	REF	REF	REF	REF
Yes	**0.79**	**0.67–0.92**	0.85	0.70–1.04
Duration of HIV infection (years)				
≤1 year	REF	REF	REF	REF
1–5 years	1.20	0.88–1.65	1.08	0.79–1.48
5–10 years	**1.56**	**1.16–2.12**	1.20	0.88–1.63
10+ years	**1.85**	**1.25–2.74**	1.21	0.80–1.83
**Behavioral**				
Age at first exchange of sex for money (years)				
<18 years	REF	REF		
≥18 years	1.14	0.82–1.60		
Use of hormonal/long-acting contraception ^a^				
No	REF	REF	REF	REF
Yes	**1.21**	**1.01–1.44**	**1.19**	**1.00–1.43**
Current relationship status				
Steady partner, co habituating	REF	REF		
Steady partner, living apart	1.16	0.94–1.44		
Single	1.02	0.83–1.26		
Alcohol use (5+ daily drinks ≥2x weekly)				
No	REF	REF		
Yes	0.93	0.81–1.08		
Drug use in prior 30 days ^b^				
No	REF	REF	REF	REF
Yes	**0.66**	**0.56–0.79**	0.92	0.76–1.12
**SEXUAL NETWORKS**				
Consistent condom use with new clients in prior 30 days				
No	REF	REF		
Yes	1.11	0.93–1.34		
No new clients in prior 30 days	1.12	0.60–1.90		
Consistent condom use with regular clients in prior 30 days				
No	REF	REF		
Yes	1.10	0.92–1.31		
No regular clients in prior 30 days	0.85	0.53–1.37		
Consistent condom use with nonpaying partners in prior 30 days				
No	REF	REF		
Yes	1.05	0.80–1.38		
No nonpaying partner in the prior 30 days	0.95	0.79–1.14		
Sex work frequency				
<20 days per month	REF	REF	REF	REF
≥20 days per month	**0.73**	**0.63–0.84**	0.91	0.75–1.10
**WORK AND SOCIAL ENVIRONMENTS**				
Primary sex work venue				
Outdoor	REF	REF		
Indoor	0.97	0.80–1.18		
**Forced shared earning from sex work**				
No	REF	REF		
Yes	0.95	0.64–1.43		
Carry condoms during sex work				
No	REF	REF		
Yes	1.01	0.85–1.20		
History of physical violence				
No	REF	REF	REF	REF
Yes	**0.79**	**0.66–0.93**	0.99	0.82–1.20
History of sexual violence				
No	REF	REF	REF	REF
Yes	**0.71**	**0.59–0.85**	0.87	0.71–1.08
Perceived safety in daily life				
No safety	REF	REF	REF	REF
Some degree of safety	1.17	0.99–1.40	1.05	0.88–1.26
**MACROSTRUCTURAL**				
Experienced lack of housing in prior 6 months				
Housed	REF	REF	REF	REF
Unhoused	**0.61**	**0.49–0.75**	**0.72**	**0.56–0.91**
Household residents				
≤4 residents	REF	REF	REF	REF
>4 residents	**0.84**	**0.72–0.97**	0.94	0.78–1.15
**Enough money to meet needs**				
Not at all	REF	REF		
A little to completely	1.04	0.87–1.24		
Number of dependents financially supported by sex work				
≤2 dependents	REF	REF	REF	REF
>2 dependents	**1.40**	**1.18–1.67**	1.10	0.92–1.33
Mobility outside of Durban ≥1x in prior 6 months				
No	REF	REF		
Yes	0.98	0.75–1.29		
Anticipated HIV-related stigma				
No	REF	REF		
Yes	0.76	0.54–1.08		
Anticipated sex work-related stigma				
No	REF	REF	REF	REF
Yes	**0.80**	**0.66–0.96**	0.87	0.72–1.06
Enacted HIV-related stigma				
No	REF	REF		
Yes	0.94	0.74–1.18		
Enacted sex work-related stigma				
No	REF	REF		
Yes	0.92	0.77–1.10		

^a^ Hormonal / long-acting contraception includes birth control pill, intrauterine device, injectable birth control, implant

^b^ Drug use includes marijuana, cocaine, crack, methamphetamine, ecstasy, heroin, and heroin derivatives.

## Discussion

South Africa’s National Strategic Plan (NSP) for HIV, TB, and STI 2023–2028 aims to reduce inequalities in access to and benefit from HIV treatment and care services for people living with HIV [55]. Sex workers are identified as a key population of focus within the NSP, and this study reinforces this mission, characterizing the high unmet treatment needs among female sex workers living with HIV in eThekwini, South Africa. In this large study among FSW living with HIV and aware of their HIV status, 65% were on treatment and 38% were virally suppressed (50% were virally suppressed among FSW currently on ART). While these clinical outcomes are suboptimal, data from this study are not far from what have been observed in a respondent driven sampling (RDS) study among FSW on treatment in eThekwini, which had a higher threshold for viral suppression (53% of FSW aware of status were virally suppressed, as defined as <1000 copies/mL) [56], and our data reflect program service users. Another RDS across 12 sites in South Africa found 59% of FSW living with HIV overall were virally suppressed (as defined as <1000 copies/mL) [33]. Further, despite low viral suppression and high marginalization among all FSW included in this study, our results further demonstrate that FSW non-virally suppressed tended to be more marginalized than those living with HIV and virally suppressed in many ways. Results reinforce high heterogeneity of vulnerability even among marginalized populations and reinforce the need for tailored services.

This study found that younger women were less likely to be virally suppressed, which has been well characterized in the literature [33, 57–59], highlighting the importance of identifying the needs of young FSW and better tailoring treatment to their needs. Viral suppression among FSW on a long-acting or hormonal contraceptive method was 19% higher than among those not using hormonal contraception. Contraception use among the overall cohort was low (35%), in comparison to sexually active women across South Africa more broadly (64%) [60], but higher than seen from programmatic data among sex workers living with and without HIV in Johannesburg and Pretoria (15%) [61]. Coupling reproductive and HIV care may support reproductive health optimization and prevention of vertical HIV transmission, but these data suggest that further exploration with longitudinal data is necessary to assess whether higher hormonal/long-acting contraception use suggests greater HIV engagement or vice versa.

Further, we found a bivariate association between recent drug use and lower viral suppression. While these findings build on existing literature [62–67], the association did not hold within the multivariable model. Illicit substance use was prevalent, as seen among FSW and people living with HIV elsewhere [11, 12, 66, 68, 69], and ART interruptions among FSW have been found to be higher among women who used any illicit drugs [69]. Qualitative research among FSW in the region provided insights into why FSW use drugs (i.e., to meet client demands and cope with unmet mental health needs) and how drug use challenged ART adherence (i.e., affecting ability to remember to attend scheduled clinic visits and take treatment on a daily basis) [17]. Therefore, among FSW living with HIV, interventions aimed at reducing and managing substance use may encourage care engagement and optimal treatment outcomes.

In addition to individual-level factors [18–24], macrostructural-level factors have increasingly been recognized for their centrality in HIV care engagement and viral suppression [8, 25–31]. A third of Siyaphambili participants reported unstable housing and we found a lack of housing to be associated with lower viral suppression. The impact of housing instability on treatment adherence and viral suppression has been documented in other settings [70], although evidence is limited within sub-Saharan Africa [63]. A longitudinal cohort of sex workers in Vancouver, Canada, similarly found a negative correlation between a lack of housing and viral suppression [34], and a systematic review of 152 studies (largely within the United States and Canada) found almost unanimously that worse housing status was associated with decreased access to and engagement with HIV services, challenges with treatment adherence, and suboptimal HIV clinical outcomes [70]. Housing instability results from complex, interrelated multi-level determinants of health. Interventions supporting stable housing may have multifaceted impacts for HIV treatment access, storage, and daily adherence, ultimately leading to optimal health outcomes and viral suppression. Finally, a complementary analysis among FSW at enrollment into Siyaphambili also reinforced the importance of structural determinants, finding that drug use, violence, stigma, and lack of ART use all contributed to lower health-related quality of life scores [71]. Taken together these multilevel determinants iteratively shape HIV transmission dynamics and heterogeneously impact FSW living with HIV, disproportionally affecting women at increased vulnerability [25, 26].

This analysis has several limitations that should be considered. Firstly, the baseline data are cross-sectional and causality between the multilevel factors and viral suppression cannot be inferred; similarly, time-varying factors like relationship status, alcohol and drug use, and housing were only accounted for at one point in time in this analysis. Thus, the associations presented may not reflect the influence of these risk factors on viral suppression over time. There were challenge categorizing variables across levels within the conceptual framework, but our categorization aligned with the existing literature, theory, and Shannon et. al’s Framework [26]. Moreover, survey items were self-reported and, therefore, may be vulnerable to reporting or social desirability biases, although associations between multilevel factors and viral suppression found here align with existent literature [11, 58, 63, 67, 70]. Additionally, we recruited in partnership with the ongoing TB HIV Care program in eThekwini. Therefore, it is possible that FSW in the area who are not engaged with the program or operating at sex work venues known by the program were less likely to be enrolled. Thus, those enrolled may be more or less vulnerable than FSW not recruited. However, given the high vulnerability among this large cohort of FSW, clearly the TB HIV Care program is reaching those in need of critical services and differentiated care to meet these unmet needs is warranted. Finally, we utilized a stringent definition of viral suppression (<50 copies/mL), but this was in alignment with the South African national guidelines [37, 41].

## Conclusion

This analysis assessed viral suppression and marginalization among a large cohort of FSW living with HIV. Unmet HIV treatment needs were substantial, with just 38% of previously diagnosed FSW living with HIV virally suppressed, and a lack of housing, substance use, and history of violence were common. Younger age and a lack of housing, in particular, were markers for greater unmet HIV treatment needs. Expanded implementation strategies and differentiated service delivery models are urgently needed to meet these needs. Future research is needed to assess the influence of these factors longitudinally and to identify effective strategies that are adapted appropriately, acceptable to sex workers, and feasible to implement.

## Supporting information

S1 Fig Modified structural determinants framework for HIV and sex work for the Siyaphambili study among female sex workers in eThekwini, South Africa. (TIF)

S2 Fig Reasons for ineligibility among screened women for participation in the Siyaphambili study between June 2018 and March 2020 in eThekwini, South Africa (n = 242). (TIF)
