# Supplementary figures and images for: ART coverage and viral suppression among female sex workers living with HIV in eThekwini, South Africa: Baseline findings from the Siyaphambili study
Source: PLOS Glob Public Health. 2024 May 22;4(5):e0002783. doi: 10.1371/journal.pgph.0002783 (PMC11111033; doi:10.1371/journal.pgph.0002783)

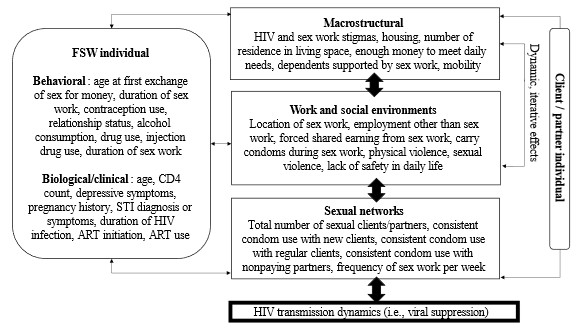

Supplement: S1 Fig — (TIF) [file pgph.0002783.s001.tif]

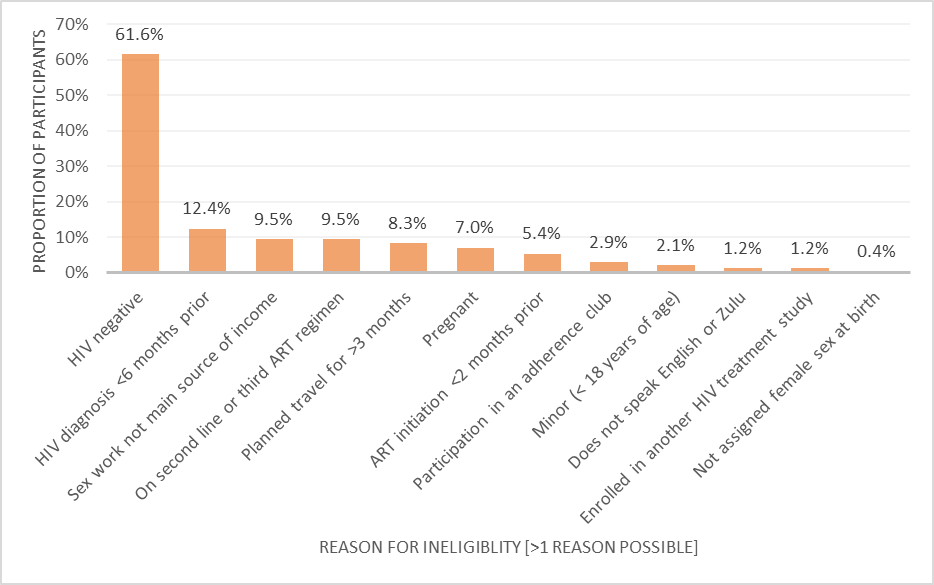

Supplement: S2 Fig — (TIF) [file pgph.0002783.s002.tif]
